# Supplementary material for: Microwave-Assisted One-Pot Lipid Extraction and Glycolipid Production from Oleaginous Yeast Saitozyma podzolica in Sugar Alcohol-Based Media
Source: Molecules. 2021 Jan 18;26(2):470. doi: 10.3390/molecules26020470 (PMC7829979; doi:10.3390/molecules26020470)
Supplement: Supplementary file 1 [file molecules-26-00470-s001.zip › Supplementary/Supplementary Figure S1.docx]

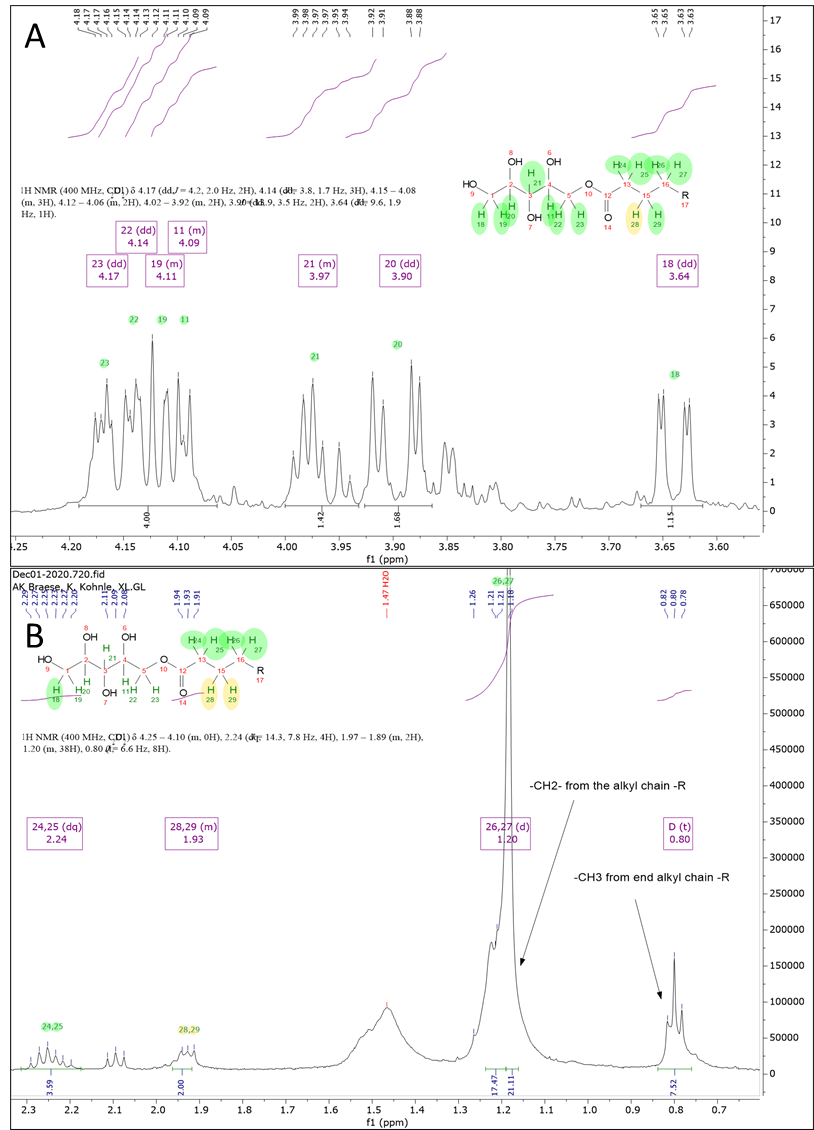


Supplementary Figure S1 Treated ^1^H-NMR experiment with zooms on the chemical shifts corresponding to the xylitol (A) and the fatty acids (B)
