# Supplementary material for: Microwave-Assisted One-Pot Lipid Extraction and Glycolipid Production from Oleaginous Yeast Saitozyma podzolica in Sugar Alcohol-Based Media
Source: Molecules. 2021 Jan 18;26(2):470. doi: 10.3390/molecules26020470 (PMC7829979; doi:10.3390/molecules26020470)
Supplement: Supplementary file 1 [file molecules-26-00470-s001.zip › Supplementary/Supplementary Figure S5.docx]

Supplementary Figure S5 Example of a GC-chromatogram presenting main FAMEs in the lipid mixture and their signal integration.
